# Commentary: Immuno-inflammatory signature for predicting therapeutic response and survival after stereotactic radiosurgery in NSCLC patients with brain metastases: a retrospective cohort study

**DOI:** 10.3389/fimmu.2026.1782399

**Published:** 2026-02-06

**Authors:** Kunpeng Yang, Wenjuan Sun, Bao Wang

**Affiliations:** 1 School of Medicine, Changchun University of Chinese Medicine, Changchun, China; 2 Department of Thoracic Oncology Surgery II, Jilin Cancer Hospital, Changchun, Jilin, China

**Keywords:** brain, edema index, immunocore, metastases, neutrophil-to-lymphocyte ratio, NSCLC, precision medicine, radiosurgery (SRS)

## Introduction

Brain metastases (BM) represent a critical evolutionary milestone in non-small cell lung cancer (NSCLC), profoundly dictating both morbidity and overall survival (OS). While stereotactic radiosurgery (SRS) is currently the standard of care for oligometastatic disease, patient responses remain highly heterogeneous. In a recent study published in Frontiers in Immunology, Zhao et al. developed a novel nomogram and decision-tree model using a cohort of 464 patients (1). Their research integrates systemic inflammation—specifically the neutrophil-to-lymphocyte ratio (NLR)—with local radiological features, such as the edema index (EI), to predict therapeutic response and OS (1). Although this integration of immunology and radiology is timely, the model’s applicability in the era of precision oncology is challenged by the static nature of its biomarkers and an underexplored weighting of molecular subtypes. This commentary seeks to refine these findings by examining them through the lens of evolving targeted therapies and dynamic immune monitoring.

## The “seed” and the treatment context: reevaluating EGFR/ALK status

A striking observation in the study by Zhao et al. is that EGFR and ALK mutation status were not identified as independent prognostic factors for OS (1). This finding contradicts the widely accepted Lung-molGPA index, in which these driver mutations are heavily weighted as positive prognostic markers (2). It is argued here that this discrepancy likely arises from a lack of stratification regarding the generation of tyrosine kinase inhibitors (TKIs) administered post-SRS. The study cohort spanned 2016 to 2022, a period of transition in clinical standards. While first-generation TKIs exhibit limited blood-brain barrier penetration, third-generation agents like osimertinib have demonstrated superior intracranial efficacy in trials such as FLAURA (3, 4). If a significant portion of the cohort received older TKIs, the survival advantage typically conferred by these mutations may have been obscured. Therefore, future models must incorporate “post-SRS systemic therapy” as a time-dependent covariate to reflect the modern therapeutic landscape accurately.

## Systemic inflammation: from snapshot to motion picture

Zhao et al. utilized pre-treatment NLR as a key node for predicting local control (1). Although valuable, a baseline “snapshot” may oversimplify the dynamic interactions between the immune system and therapeutic interventions. SRS is capable of inducing immunogenic cell death (ICD), which releases damage-associated molecular patterns (DAMPs) to prime systemic anti-tumor immunity. Consequently, the dynamic change in NLR (dNLR)—particularly an early post-treatment reduction—is often a more robust predictor of survival than baseline values (5). A persistent or rising NLR post-SRS may indicate radioresistance or a failure to trigger an adaptive immune response, thereby identifying patients who require treatment intensification.

## The edema index: a proxy for the “cold” tumor microenvironment?

**Figure 1:**
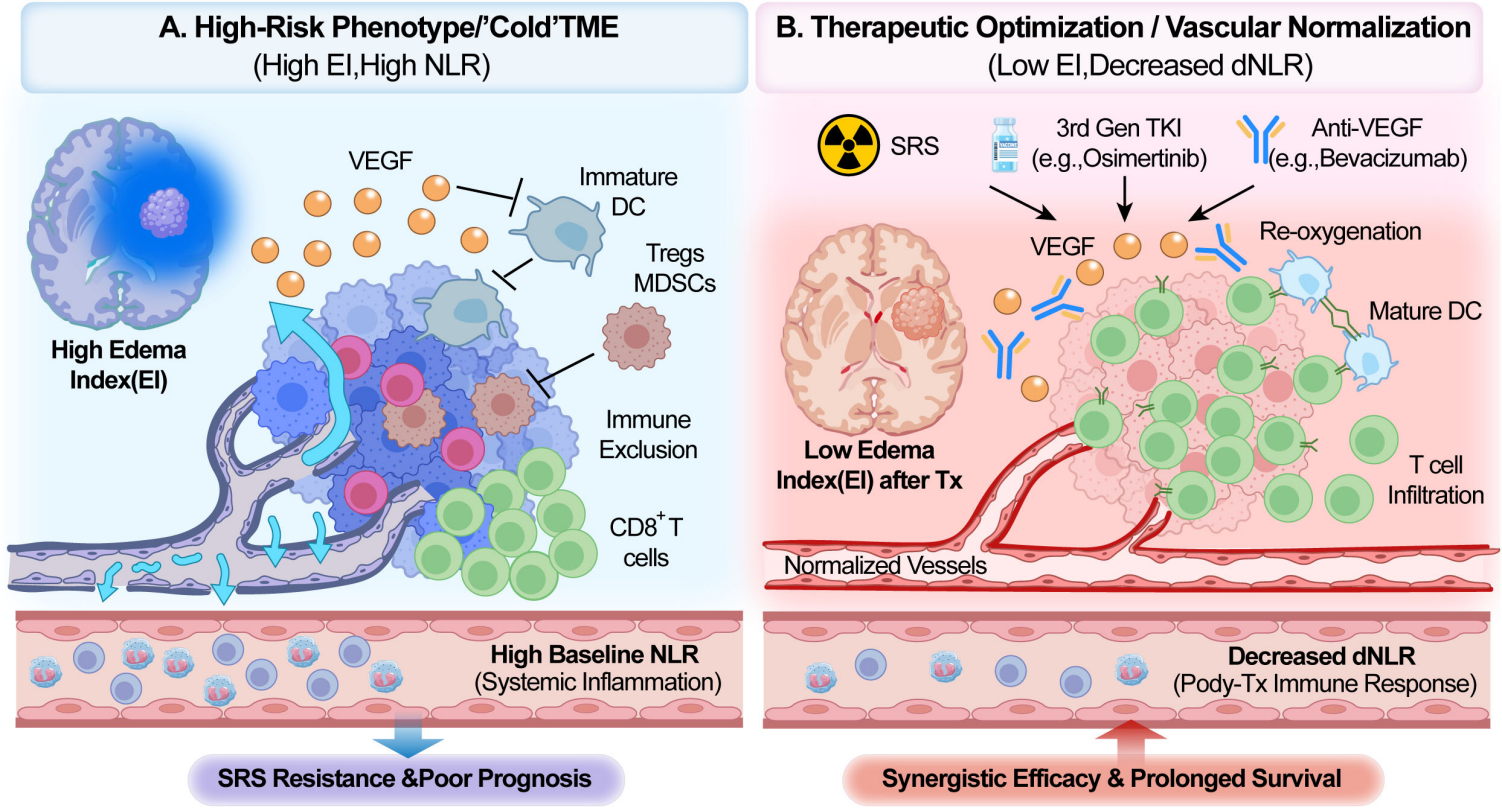
The immuno-radiological axis in NsCLC brain metastases: mechanisms and therapeutic implications. **(A)** High-Risk Phenotype/”Cold” Tumor Microenvironment (High EI, High Baseline NLR): A “cold” tumor microenvironment characterized by poor prognosis is presented. At the macroscopic level, MRI reveals significant peritumoral edema, which indicates a high edema index (EI). At the microscopic level, this condition is driven by the extensive secretion of vascular endothelial growth factor (VEGF) by the tumor, resulting in disordered vascular structures and hyperpermeable leakage. Elevated VEGF levels simultaneously exert potent immunosuppressive effects. Specifically, the maturation of dendritic cells (DCs) is inhibited, while regulatory T cells (Tregs) and myeloid-derived suppressor cells (MDSCs) are recruited. Consequently, effector CD8+ T cells are excluded from the tumor core, a process termed immune exclusion. At the systemic level, increased neutrophil counts and decreased lymphocyte counts are observed within the vasculature, reflected by a high baseline neutrophil-to-lymphocyte ratio (NLR). This biological state results in resistance to stereotactic radiosurgery (SRS) and poor clinical outcomes. **(B)** Therapeutic optimization/vascular normalization (low EI, decreased dNLR): an optimized strategy is demonstrated through the integration of SRS, third-generation tyrosine kinase inhibitors with high intracranial penetration (e.g., osimertinib), and anti-VEGF therapy (e.g., bevacizumab). Anti-VEGF agents neutralize excess VEGF, thereby promoting vascular normalization. This process not only significantly alleviates edema, as shown by a low post-treatment EI on MRI, but also improves tumor re-oxygenation. Enhanced oxygenation subsequently increases radiosensitivity. The alleviation of immunosuppression allows DCs to reach maturity and successfully guide the infiltration of CD8+ T cells into the tumor. This transition converts a “cold” tumor into an immunologically active “hot” tumor. Systemically, successful immune activation is evidenced by a recovery in lymphocyte levels, resulting in a decreased dynamic NLR (dNLR). Ultimately, this combined strategy achieves synergistic efficacy and prolonged patient survival. NSCLC, non-small cell lung cancer; TME, tumor microenvironment; EI, edema index; NLR, neutrophil-to-lymphocyte ratio; dNLR, dynamic NLR; VEGF, vascular endothelial growth factor; DC, dendritic cell; Treg, regulatory T cell; MDSC, myeloid-derived suppressor cell; SRS, stereotactic radiosurgery; TKI, tyrosine kinase inhibitor; Tx, treatment.

The identification of the Edema Index (EI) as an independent prognostic factor is a significant highlight of the original study (1). Beyond its physical mass effect, peritumoral edema represents a specific immunosuppressive state within the tumor microenvironment (TME). Vasogenic edema is primarily driven by vascular endothelial growth factor (VEGF) and the subsequent disruption of vascular integrity (6). High VEGF levels induce profound immunosuppression by inhibiting dendritic cell (DC) maturation and promoting the infiltration of regulatory T cells (7). Thus, a high EI may serve as a radiological surrogate for a VEGF-rich, “cold” TME that is resistant to immune-mediated clearance (**Figure 1A**). This rationale supports the combination of anti-angiogenic agents, such as bevacizumab, with SRS. Such agents not only manage edema but also “normalize” tumor vasculature, improving oxygenation and converting an immunosuppressive TME into an immunostimulatory one (**Figure 1B**) (8).

## Conclusion and future perspectives

The research conducted by Zhao et al. provides a pragmatic tool for risk stratification in NSCLC patients with brain metastases (1). However, achieving true precision medicine necessitates a shift from static biomarkers to multi-dimensional dynamic profiles. It is proposed that future iterations of these models should reintegrate molecular status weighted by the intracranial potency of the drugs used, adopt dynamic metrics such as dNLR to capture real-time immune responses, and investigate EI as a targetable biomarker for vascular-normalizing therapies. Validating these expanded signatures within prospective cohorts will be essential to fully unlocking the potential of comprehensive brain metastasis management.
